# Management of the Wheat Curl Mite and Wheat Streak Mosaic Virus With Insecticides on Spring and Winter Wheat

**DOI:** 10.3389/fpls.2021.682631

**Published:** 2021-06-08

**Authors:** Carmen Y. Murphy, Mary E. Burrows

**Affiliations:** Plant Sciences and Plant Pathology Department, Montana State University, Bozeman, MT, United States

**Keywords:** eriophyid, mite, wheat, cereal, insecticide

## Abstract

The wheat curl mite (WCM, *Aceria tosichella*, Keifer) is an eriophyid mite species complex that causes damage to cereal crops in the Northern Great Plains by feeding damage and through the transmission of plant viruses, such as wheat streak mosaic virus. Insecticide treatments were evaluated in the greenhouse and field for efficacy at managing the WCM complex on wheat. Treatments tested were carbamates, organophosphates, pyrethroids, a neonicotinoid seed treatment, mite growth inhibitors, and Organic Materials Review Institute–approved biocontrols, soaps, and oils. Treatment with carbamates, organophosphates, and pyrethroids decreased WCM in greenhouse trials compared with untreated controls 14 days after infestation. The seed treatment, mite growth inhibitors, and organic pesticides did not reduce WCM populations effectively and consistently. The timing of application was tested using a sulfur solution as the experimental treatment. Treating plants with sulfur seven days after mite infestation reduced mites compared with the untreated control. In contrast, prophylactically applied sulfur and sulfur applied 14 days after mite infestation were not effective. When tested under field conditions with plots infested with viruliferous mites, there was no yield difference detected between untreated control plots and plots sprayed with insecticides. Select carbamates, organophosphates, and pyrethroids have a potential for use in greenhouse mite management when appropriate.

## Introduction

The wheat curl mite (WCM, *Aceria tosichella*, Keifer) is an eriophyid mite species complex that causes feeding damage to cereal crops (Harvey et al., 2000) and vectors a number of viral pathogens, such as wheat streak mosaic virus (WSMV), triticum mosaic virus, High Plains wheat mosaic virus, and Brome streak mosaic virus (Slykhuis, 1955; Seifers et al., 1997, 2009; Stephan et al., 2008). Economical losses to these cereal viruses have been reported in epidemics since the 1950s in Montana (Brey et al., 1998) and other states in the Northern Great Plains region of the United States (Burrows et al., 2015). Infection with WSMV alone can be highly damaging to cereal crops, and research indicates that viruses in tandem can compound damage (Tatineni et al., 2010; Byamukama et al., 2014). WSMV is a member of the *Potyviridae* family (Stenger et al., 1998), and symptoms include a yellow mosaic on leaves, plant stunting, decreased grain yield, and plant death (Staples and Allington, 1956). Management recommendations include removing the “green bridge” plants that can allow mites to overwinter and oversummer, such as grassy weeds and volunteer wheat (Thomas and Hein, 2003; Jiang et al., 2005), altering planting date to avoid the mite vector (Hunger et al., 1992), and sources of plant tolerance or resistance to the mite or viruses (Martin et al., 1984; Price et al., 2014).

The difficulty of managing the WCM species complex can be partially attributed to the variability in genetic lineages within the complex that spans a large plant host range (Skoracka et al., 2013), with 90 host species recorded (Skoracka et al., 2010). The WCM species complex can therefore survive on multiple grass species and move by wind currents to re-infest in subsequent cropping seasons by surviving on weeds, alternative hosts, and winter wheat germinating in the fall (Coutts et al., 2008; Ito et al., 2012; Ranabhat et al., 2018).

Insecticides and acaricides are often used to curtail eriophyid mites on high value cash crops, such as citrus, coconuts, olives, and others (Kalaisekar et al., 2000; Pushpa and Nandihalli, 2008). Using chemicals to treat the WCM, and by proxy the viruses it vectors, is less feasible for cereal crops because of the low input and low return nature of production. An option that has been used for plant pest management since the early days of agriculture is sulfur (Tweedy, 1981), and researchers have used it to dust plants or in a foliar solution for greenhouse WCM management (Jeppson et al., 1975; Conner et al., 1991; Li et al., 2007).

A number of synthetic insecticides have also been tested for management of the WCM complex, such as carbamate carbofuran (Harvey et al., 1979) and organophosphate terbufos (Fritts et al., 1999), which are no longer labeled for cereals because of off-target effects. Similar broad-spectrum insecticides run the risk of reducing natural mite predators in the field (Stilwell, 2009) and beneficial organisms like pollinators (Moore et al., 1998; Nash et al., 2008). There are also concerns for human health, causing some insecticides to be put under regulatory review. A relevant example is chlorpyrifos (Racke, 1993; Ventura et al., 2019). The goal of this study was to evaluate the efficacy of insecticide treatments on the WCM complex in the greenhouse under controlled conditions and in field studies, with varying modes of action tested. Included were synthetic products and Organic Materials Review Institute (OMRI)-approved products for organic cereal growers.

## Materials and Methods

### Wheat Curl Mite Population

Mites were sourced from a colony established from WCM collected from the Arthur H. Post Agronomy Research Farm, Bozeman, MT, as described by Ito et al. (2012). Other mite populations collected from this region clustered with North American “Type 1” WCM; however, the mite biotype used in this research was not evaluated. The colony was maintained in a plant growth chamber at 18–24°C. Mite populations were raised on spring wheat cultivar Choteau, in MSU mix soil [1:1:1 Canadian sphagnum peat moss, mineral soil mix, and Aquagro 2000G (Aquatrols, Paulsboro, NJ, United States)].

### Greenhouse Insecticide Efficacy Trials

Twenty treatments (Table 1) were analyzed in the greenhouse for efficacy against WCM-infested spring wheat plants (*Triticum aestivum* L. cv. Choteau). Experiments were conducted in the Plant Growth Center at Montana State University. Due to the large number of treatments tested and the time-consuming nature of mite counting, treatments were grouped by mode of action and tested in four separate experiments. Each experiment was performed three times and included a mite-infested control without insecticide. Experiments were organized as a completely randomized design with eight replications per treatment. Wheat seeds were planted in MSU mix (described above) in 12.7 × 9 cm pots at conditions of 24 ± 4°C (16 h day) and 18 ± 4°C (8 h night), watered daily or as necessary, and thinned to one plant, the experimental unit. Wheat seeds were coated with thiamethoxam prior to planting, courtesy of Syngenta®.

**Table 1:** Products selected for WCM insecticide efficacy trials.

**Class-MOA^**y**^**	**Active ingredient/s**	**Trade name/Manufacturer**	**Active ingredient amt**	**ExpID^**z**^**
Carbamate-1A	Methomyl	Lannate LV/Dupont	163 g a.i./ha	A
Carbamate-1A	Aldicarb	Temik/Bayer	185 g a.i./ha	A
Organophosphate-1B	Dimethoate	Dimethoate 400/Loveland products	439 g a.i./ha	A
Organophosphate-1B	Chlorpyrifos	Lorsban 4E/Dow	526 g a.i./ha	A
Pyrethroid-3A	Zeta-cypermethrin	Mustang Maxx/FMC	27 g a.i./ha	A
Pyrethroid-3A	Lambda-cyhalothrin	Warrior II/Syngenta	34 g a.i./ha	A
Mite growth inhibitor-10A	Hexythiazox	Onager/Gowan	156 g a.i./ha	B
Mite growth inhibitor-10B	Etoxazole	Zeal/Valent	157 g a.i./ha	B
Organic biocontrol	*Bacillus mycoides* J	BmJ/Montana microbial products	1.4*10^10^ spores/ha	B
Organic pesticide	Petroleum distillate	Sunspray/Sunoco	4,883 g a.i./ha	B
Organic pesticide	Extract of neem oil	Trilogy/Certis	3,460 g a.i./ha	B
Organic pesticide	Chitosan	Chitosan/Sigma Aldrich	825 g a.i./ha	C
Organic pesticide	Neem oil Azadirachtin	Debug turbo/Agro logistic	130 g a.i./ha 1.4 g a.i./ha	C
Organic pesticide	Potassium salts of fatty acids	Des-X/Certis USA	2,323 g a.i./ha	C
Organic pesticide	*Isaria fumosorosea* Apopka strain 97	PFR-97/Certis USA	449 g a.i./ha	C
Organic pesticide	Potassium silicate	Sil-matrix/Certis USA	717 g a.i./ha	C
Organic pesticide	Sulfur	Sulfur/Planet Natural	1,156 g a.i./ha	C
•Neonicotinoid-4A DMI Fungicide-G1 PA Fungicide-A1	Thiamethoxam Difenoconazole Mefenoxam	Cruiser Maxx® Cereals seed treatment/Syngenta	272 g a.i./ha 327 g a.i./ha 54 g a.i./ha	D

y *Mode of action*.

z *Experiment identification letter*.

Wheat curl mites were transferred to experimental plants from the growth chamber colony at growth stage DC 13–14 (Zadoks et al., 1974) by attaching infested leaves (6 cm in length with 30 WCMs) using paper clips to the ligule end and adaxial surface of the youngest expanded leaf to allow mites to move from the leaf piece to the experimental plant. After 2 days, leaf pieces and paper clips were removed from plants, and the number of mites remaining on the leaf piece was counted to estimate the approximate number of mites out of the 30 WCMs applied that transferred to the experimental plant. At the time of WCM transfer, plants were enclosed in a plastic cover [14 cm height, 8.5 cm diameter, Pro-Kal containers (Fabri-Kal, Kalamazoo, MI, United States)] with three cutouts (5 × 2 cm) sealed with a fine nylon lab pak mesh (25 μm; Sefar AG, Heiden, Switzerland) to prevent mite spread between plants. Pots were positioned eight per tray into black plastic 1,020 flats with no holes, for ease of watering due to plant covers.

Foliar treatments were applied 14 days after infestation (DAI) using a spray booth (Generation III Research Sprayer, DeVries Manufacturing, Hollandale, MN, United States) calibrated to run at 3.78 kph and spray a length of 1.8 m and at a pressure of 275 kPa with nozzle height 35 cm above the top of the plant. An 8002vs nozzle was used for application, manufactured by TeeJet® (Springfield, IL), and pesticides were prepared and mixed with water to make 500 ml of solution. Active ingredient concentrations were the highest labeled rates acceptable for wheat. The granular aldicarb treatment was applied to the soil and watered as per label directions. Three to four days after treatment, whole plant mite number was calculated by counting mites under a dissecting microscope at 10× magnification. Mites were determined to be living based on movement, opacity, and white color.

To account for the fact that plants did not all have the same number of mites transferred initially, a population growth rate was calculated to determine how populations responded to treatments. This calculation used the Malthusian population growth model *P(t)* = *P*_0_*e*^rt^ given that P_0_ is the number of mites initially transferred to experimental plants, P(t) is the total plant number of mites at experiment end, “r” is the population growth rate, and “t” is time (in days) that mites were on plants (Yaninek et al., 1989). For ease of understanding differences in treatments, as population growth rate can be difficult to comprehend, population growth rate was then used to calculate adjusted total WCM, with the equation *P(t)* = *30e*^r14^ for the eight replicates, from which an average was calculated. This equation gives an estimated total mite number if all pots had exactly 30 mites transferred to start with, held constant at 14 days.

Sulfur was used in a treatment timing experiment, repeated twice. Foliar sulfur treatments were applied using the spray booth, calibrated as above. Treatments were spraying sulfur prophylactically 1 day prior to mite application, 7 DAI, and 14 DAI.

### Statistical Analysis of Greenhouse Trials

Data from all three trials were not combined because of contamination with a morphologically similar mite, the cereal rust mite (*Abacarus hystrix*), in the final trial repetition. A linear model with treatment as a fixed effect and total WCM number as the response variable was run using R software version 3.4.3. A Tukey test was conducted using the “multcomp” package (Hothorn et al., 2008), and mean comparison letters were generated. The assumption of normality was checked using Q–Q plots and the assumption of homogeneity of variances with standardized residual plots.

### Effects of Insecticides on Viruliferous Mite Inoculated Field Plots

Field trials were conducted on spring wheat (*Tritcum aestivum* cv. Choteau) in 2013 and winter wheat (cv. Genou) in 2014 at the Lutz Farm in Bozeman, Montana (45°48′38.5″N, 111°03′03.1″W, elevation 1,409 m). The soil at the farm is black dog silt loam, with an approximate pH of 7.3. The selected field design was a randomized complete block with seven blocks, with a total of 70 plots (2.75 × 2.45 m) of eight rows with 60 wheat seeds/m^2^, with buffer regions of 2 m between plots. Buffer strips of wheat were grown on the edge of field plots to check for natural WCM presence. Buffer zones were checked for natural WCM presence and mite movement from experimental plots twice in the growing season by sampling 20 leaves, selected randomly. WCMs were not detected in buffer zones in both years.

Field plots were inoculated in spring with WCM viruliferous for WSMV at growth stage DC 21 (Zadoks et al., 1974). WCMs were grown on spring wheat cv. Choteau that had been mechanically inoculated with an isolate of WSMV designated “Conrad-I” as described in Ito et al. (2012). Winter wheat plots were not inoculated in the fall. Plots were infested with 150 mites each from the source population used in greenhouse trials (described above), by attaching leaf pieces to one centrally located plant per plot.

Treatments were applied using a CO_2_-charged backpack sprayer with 4, 8,002 VS nozzles by TeeJet® (Springfield, IL) spaced one foot apart 18 days after mite infestation, at DC 39 (Zadoks et al., 1974). Ten treatments, including an inoculated but untreated control (sprayed with water), were randomized in seven blocks. Treatments selected were dimethoate, chlorpyrifos, zeta-cypermethrin, lambda-cyhalothrin, hexythiazox, neem oil with azadirachtin, *Isaria fumosorosea* Apopka, potassium salts, and sulfur using the same active ingredient amount as described for greenhouse trials (Table 1). In 2014, eight treatments were the same, while methomyl and etoxazole replaced *I. fumosorosea* Apopka and sulfur treatments because of the difficulty in the application of these products with the spray boom in the previous year. Sampling for WCM and WSMV was conducted 14 days posttreatment. Thirty fully expanded leaves were collected per plot in a systematic manner from the inner 1.75 m and inner six rows and stored on ice in 10.2 × 15.2 cm plastic sample bags (Fisher Scientific, Pittsburgh, PA, United States). Leaves were checked for mite presence under a dissecting microscope at a magnification of 10×. The leaves were then frozen at −20°C until virus detection with ELISA, conducted using the methodology described in Ito et al. (2012). At wheat maturity plots were harvested using a research combine (Wintersteiger, Salt LakeCity, UT, United States), and samples were returned to Montana State University to be dried and cleaned. Whole plot yield was calculated from grain weight using plot lengths taken on the day of harvest. Samples were also analyzed for moisture, test weight, and protein content at the MSU cereal quality laboratory.

### Statistical Analysis of Field Trials

Yield was the selected response variable using a mixed-effects model with the “lme” function in the “nlme” package in R version 4.0.2 (Pinheiro et al., 2021). The fixed effects included in the model were WCM per plot, virus presence, and insecticide treatment. Block was included in the model as a random effect. WSMV infection at the plot scale was calculated as a proportion of sampled leaves testing positive for WSMV, and logit transformed to meet model assumptions, with untransformed data displayed. Residuals for normality of the model were checked using Q–Q plots, and the final model was selected in a sequential removal of components from a full model that checked interaction terms between factors. No interactions were found between factors, so interaction terms were excluded from the final statistical model.

## Results

In greenhouse trials, the carbamates, organophosphates, and pyrethroids tested reduced the total WCM number on plants compared to untreated control plants (*p* < 0.001) (Table 2). Active ingredients aldicarb, chlorpyrifos, and methomyl were more effective at reducing WCM than dimethoate.

**Table 2:** Effect of carbamate, organophosphate, and pyrethroid treatments on average WCM total per plant.

**Active ingredient/s**	**Trade name**	**Adj. avg number of WCM^**x**^**	**SE^**y**^**	**MCL^**z**^**
Untreated control	Untreated control	132	18	c
Aldicarb	Temik	2	1	a
Chlorpyrifos	Lorsban 4E	11	3	a
Methomyl	Lannate LV	21	6	a
Lambda-cyhalothrin	Warrior II	42	13	ab
Zeta-cypermethrin	Mustang Maxx	47	15	ab
Dimethoate	Dimethoate 400	73	14	b

x *Calculated using the population growth rate of WCM on each experimental unit and an average taken of adjusted WCM using the formula *P(t)* = *30e*^r14^*.

y *Standard error of the mean*.

z *Mean comparison letter from Tukey's HSD, means that are not different at *a* = 0.05 indicated by the same letter*.

The mite growth inhibitors, organic biocontrol, and organic pesticides did not reduce wheat curl mite numbers when applied 14 DAI under greenhouse conditions (Table 3).

**Table 3:** Effect of mite growth inhibitor, organic biocontrol, and organic pesticide treatments on average WCM total per plant.

**Active ingredient/s**	**Trade name**	**Adj. avg number of WCM^**x**^**	**SE^**y**^**	**MCL^a^**
Untreated control	Untreated control	98	14	a
Hexythiazox	Onager	79	19	a
Etoxazole	Zeal	81	17	a
Extract of neem oil	Trilogy	98	16	a
Petroleum distillate	Sunspray	138	41	a
*Bacillus mycoides* J	BmJ	141	26	a

x *Calculated using the population growth rate of WCM on each experimental unit and an average taken of adjusted WCM using the formula P(t) = 30e^r14^*.

y *Standard error of the mean*.

z *Mean comparison letter from Tukey's HSD, means that are not different at a = 0.05 indicated by the same letter*.

Additional organic pesticides tested did not reduce the total WCM number on plants compared with untreated controls (Table 4).

**Table 4:** Effect of organic pesticide treatments on average WCM total per plant.

**Active ingredient/s**	**Trade name**	**Adj. avg number of WCM^**x**^**	**SE^**y**^**	**MCL^**z**^**
Untreated control	Untreated control	86	31	a
Potassium salts of fatty acids	Des-X	31	18	a
*Isaria fumosorosea* Apopka strain 97	PFR-97	47	10	a
Neem oil, Azadirachtin	Debug turbo	48	24	a
Chitosan	Chitosan	74	35	a
Potassium silicate	Sil-matrix	86	34	a
Sulfur	Sulfur	96	35	a

x *Calculated using the population growth rate of WCM on each experimental unit and an average taken of adjusted WCM using the formula *P(t)* = *30e*^r14^*.

y *Standard error of the mean*.

z *Mean comparison letter from Tukey's HSD, means that are not different at *a* = 0.05 indicated by the same letter*.

The seed treatment tested did not reduce mites compared with controls (Table 5).

**Table 5:** Effect of neonicotinoid seed treatment on average WCM total per plant.

**Active ingredient/s**	**Trade name**	**Adj. avg number of WCM^**x**^**	**SE^**y**^**	**MCL^**z**^**
Untreated control	Untreated control	171	52	a
Thiamethoxam Difenoconazole Mefenoxam	Cruiser Maxx^®^ Cereals	139	27	a

x *Calculated using the population growth rate of WCM on each experimental unit and an average taken of adjusted WCM using the formula *P(t)* = *30e*^r14^*.

y *Standard error of the mean*.

z *Mean comparison letter from Tukey's HSD, means that are not different at *a* = 0.05 indicated by the same letter*.

The timing of sulfur treatment had an impact on the efficacy of treatment (Table 6). There was a difference between temporal treatments with sulfur applied seven DAI reducing WCM numbers and no difference between the prophylactic treatment, the 14 DAI treatment, and the untreated control (*p* = 0.028).

**Table 6:** Effect of sulfur application timing on average WCM total per plant.

**Treatment^**x**^**	**Adj. avg number of WCM**	**SE^**y**^**	**MCL^**z**^**
Untreated control	213	27	b
Sulfur pre-treatment	174	28	ab
Sulfur 7 DAI^w^	35	7	a
Sulfur 14 DAI^w^	212	63	b

w *DAI, days after infestation. Sulfur pre-treatment applied 1 day prior to mite infestation*.

x *Calculated using the population growth rate of WCM on each experimental unit and an average taken of adjusted WCM using the formula *P(t)* = *30e*^*r14*^*.

y *Standard error of the mean*.

z *Mean comparison letter from Tukey's HSD, means that are not different at *a* = 0.05 indicated by the same letter*.

In the 2013 spring wheat field trial, there was no evidence of treatment improving yield compared to untreated controls (*p* = 0.912). Mite numbers detected per leaf were relatively low on sampled leaves (data not shown), while the presence of WSMV was easily discerned (Table 7). The presence of virus in plots did indicate an impact on yield (*p* = 0.015). In the 2014 winter wheat plots, no impact of treatment was detected on yield (*p* = 0.218), and the presence of mite and virus was much lower on the winter wheat than the year prior on spring wheat (Table 7).

**Table 7:** Wheat streak mosaic virus presence and grain yield for spring wheat and winter wheat field plots.

**Active ingredient/s**	**Proportion WSMV^**z**^ 2013**	**SE^**y**^**	**Yield (kg/ha) 2013**	**SE^**y**^**	**Proportion WSMV^**z**^ 2014**	**SE^**y**^**	**Yield (kg/ha) 2014**	**SE^**y**^**
Untreated control	0.50	0.15	1,498	125	0.03	0.03	3,033	506
Dimethoate	0.54	0.13	1,568	194	0.01	0.01	2,106	113
Chlorpyrifos	0.53	0.10	1,802	279	0.06	0.03	2,050	511
Zeta-cypermethrin	0.43	0.13	1,729	206	0.01	0.01	2,799	639
Lambda-cyhalothrin	0.61	0.10	1,640	253	0.03	0.02	2,441	466
Hexythiazox	0.52	0.15	1,676	180	0.01	0.01	1,904	224
Neem oil, Azadirachtin	0.69	0.11	1,454	114	0.03	0.03	2,443	653
*Isaria fumosorosea* Apopka strain 97	0.39	0.14	1,863	187	NA	NA	NA	NA
Potassium salts of fatty acids	0.62	0.13	1,600	150	0.02	0.01	2,597	538
Sulfur	0.59	0.13	1,432	163	NA	NA	NA	NA
Methomyl	NA	NA	NA	NA	0.01	0.01	3,402	467
Etoxazole	NA	NA	NA	NA	0.02	0.01	1,954	244

y *Standard error of the mean*.

z *Average proportion of leaves (*n* = 30) per plot infected with WSMV determined by ELISA*.

## Discussion

The aim of this study was to evaluate the efficacy of insecticides on WCM populations in controlled greenhouse conditions and in the field. Insecticides provide an additional tool in the management of other eriophyid mite species and have the potential for use in conjunction with other integrated pest management strategies. In a controlled environment, treatments effective at reducing WCM numbers on spring wheat were the organophosphate, carbamate, and pyrethroid insecticides. Active ingredients chlorpyrifos, methomyl, zeta-cypermethrin, lambda-cyhalothrin, and dimethoate were useful in managing greenhouse mite populations. These treatments are registered for use in the United States for cereals. Active ingredient aldicarb consistently reduced WCM; however, it is no longer registered for use on cereal crops in the United Sates. Chlorpyrifos, although available, is not recommended for management. This product is banned in the European Union, and its use is contested and controversial in the United Sates because of concerns with human safety.

Non-synthetics tested in this study did not reduce WCM populations at the selected rates and 14 DAI application timing. In other systems, non-synthetic options have been useful for eriophyid mite management. Neem oil is a prevalent treatment for the eriophyid coconut mite *Aceria guerreronis* on immature nuts (Pushpa and Nandihalli, 2008; Bagde et al., 2014). Demeton-S-methyl application, an organic product, was shown to reduce damage to coconuts from *A. guerreronis* in India (Muthiah and Bhaskaran, 1999). Garlic essential oil had acaricidal activity on eriophyid olive mites (Mossa et al., 2018). Compared with other eriophyid mites, management of the WCM on wheat is hindered when mite populations are high and wheat leaves curl inward under pressure, protecting WCM from the elements (Somsen and Sill, 1970). Contact foliar treatments may not be appropriate for this reason.

Under field conditions, spraying insecticides to control WCM as a method to help prevent WSMV damage and therefore prevent yield loss was not beneficial in the years these trials were run. On spring wheat plots, virus presence was relatively high and took a toll on grain yield, while mites were not easily detected in plots. In winter wheat, virus presence was too low to detect any impact of treatment for vector management and may require the addition of a fall inoculation for higher disease pressure. A previous study on Montana wheat fields indicated that spring inoculation led to higher virus presence on winter wheat than fall inoculation, with wheat cultivars differing in mite and virus tolerance (Miller et al., 2014). WCMs are dispersed by wind, and mite movement is often difficult to track, which can make scouting for the mite challenging and leads to variability in WCM presence each year. Natural WCM presence at the field site was not detected in buffer strips of wheat; however, it is possible that spring wheat plots were impacted by naturally occurring mites. WCMs have been found in the county in years prior and are spread by wind.

Timing of inoculation and timing of insecticide treatment are important factors for these studies. In the greenhouse, manipulating the timing of sulfur application revealed that treating plants infested with viruliferous mites at 7 DAI was more successful than treating at 14 DAI. Spraying earlier when mite numbers are potentially lower, and leaves are uncurled, requires regular scouting to detect the mite or virus, which may not be realistic for producers in large-scale cereal production.

Using insecticides for vector management in cereal production raises additional factors to consider. Concerns include environmental impacts of synthetic treatments, cost of treatment for producers compared with returns, target organism resistance to treatments, impact on beneficial organisms and naturally occurring mite predators (such as thrips) (Stilwell, 2009), and impacts to human health. Resistance becomes a concern when multiple sprays are needed in a season and pressure is high on target organisms. For the WCM complex, it has a high rate of reproduction and fast generational turnover that a few mites surviving after foliar spray can lead to a new generation in 10 days (Somsen and Sill, 1970). Multiple sprays may be required to see a yield benefit from treatment, which is likely not cost-effective in a cereal system, and extra treatments increase the risk for vector resistance. In addition, a taxonomic study conducted on the wheat curl mite has shown that it is likely a cryptic species complex (Skoracka et al., 2012, Skoracka et al., 2017). Two distinct WCM types exist in North America, Type 1 and Type 2, with tested Montana populations identified as Type 1 (Hein et al., 2012). It is unknown if WCM biotypes will respond differently to chemical treatments. Resistance to treatments could also differ across biotypes within the WCM complex with persistent and prolonged exposure to insecticides. In summary, greenhouse populations of WCM were reduced with select carbamates, organophosphates, and pyrethroids. Greenhouse and research facilities often deal with unwanted pests, and the WCM can be problematic in facilities where cereals are regularly propagated. This study demonstrated that select insecticides were effective at reducing WCM populations and may be useful in protecting such plants. Although efficacy was seen in greenhouse conditions, an effect of treatment was not seen in inoculated field trials. Using insecticides for WCM management in the field may not be feasible, and elucidating application timing, treatment rates, and cost analysis is required before it is considered appropriate. Therefore, it is important to consider the holistic system and evaluate the risk–reward ratio to insecticide application and other management options before incorporating an insecticide treatment into integrated mite management for management of viral pathogens.

## Data Availability Statement

The raw data supporting the conclusions of this article will be made available by the authors, without undue reservation.

## Author Contributions

MB conceived experiments, acquired funding, assisted with experimental design, and provided manuscript edits. CM conducted experiments, ran analyses, and wrote the manuscript. Both authors contributed to the article and approved the submitted version.

## Conflict of Interest

The authors declare that the research was conducted in the absence of any commercial or financial relationships that could be construed as a potential conflict of interest.
